# Prevalence and drug susceptibility pattern of *Salmonella* isolates from apparently healthy slaughter cattle and personnel working at the Jimma municipal abattoir, south-West Ethiopia

**DOI:** 10.1186/s40794-018-0072-6

**Published:** 2018-09-24

**Authors:** Samson Takele, Kifle Woldemichael, Mulatu Gashaw, Haimanot Tassew, Moti Yohannes, Alemseged Abdissa

**Affiliations:** 1 Department of Laboratory Sciences, Jimma University, Jimma, Ethiopia; 2 Department of Epidemiology, Jimma University, Jimma, Ethiopia; 3 School of Veterinary Medicine, Jimma University, Jimma, Ethiopia

**Keywords:** *Salmonella*, Antibiotic resistance, Cattle, Abattoir personnel

## Abstract

**Background:**

*Salmonella* species are among the most common food borne pathogens worldwide and their infection is one of the major global public health problems. During the last decade, multidrug resistant *Salmonella* species have greatly increased in humans and animals. So the aim of this study was to determine prevalence and antibiotic susceptibility pattern of *Salmonella* in apparently healthy slaughterer cattle and personnel working at the Jimma abattoir.

**Method:**

A cross-sectional study was conducted from May to September 2016 at the Jimma abattoir. A total of 440 samples consisting of carcass swabs (*n* = 195), cattle feces (*n* = 195), and human stool (*n* = 50) were collected. Standard isolation and identification procedures were performed to identify *Salmonella* isolates. Antimicrobial susceptibility tests were also carried out on each isolate.

**Results:**

The overall proportion of *Salmonella* positive isolates was 9.5% in all samples, of which 11.3% were from carcass swabs, 5.6% from cattle feces, and 18% from human stool. All isolates were resistant to tasted antibiotics except Ciprofloxacin.

**Conclusion:**

This study ascertains that Salmonella were widely distributed and significant proportions have developed resistance to routinely prescribed antibiotics. Therefore, there is needed to implement urgent intervention programs in study area.

## Background

Diseases caused by *Salmonella* represent an important public health problem worldwide. It is estimated that globally 93.8 million cases and 155,000 deaths are associated with gastroenteritis due to *Salmonella* species annually. Evidence indicated that 85.6% were estimated to be food borne, and infection was associated with many different food types, including beef and beef products [1, 2].

Pathogens are mainly disseminated through the animal trade and uncooked animal food products [3, 4]. The process of removing the gastrointestinal tract during meat processing is regarded as one of the most important sources of contamination of carcass and organs with *Salmonella* at abattoirs, and also the hands of abattoir employees can be the vector to spread *Salmonella* through cross contamination [5–7].

In humans, in addition to concern about foodborne disease caused by *Salmonella* species, concern was raised about the impact of acquired antimicrobial resistance transferred among these organisms, which limits therapeutic options both in veterinary and public health practice [8, 9]. Multidrug resistant (MDR) isolates were widely reported in Europe and America from travelers and adopted children [10]. Antimicrobial drug misuse, drugs prescription without susceptibility testing, self-medication, and long hospitalization were suggested as contribution to MDR in developing nations [11].

In Ethiopia, several factors including unhygienic living circumstances, tradition of raw meat consumption, and indiscriminate use of antimicrobials may substantially contribute to the occurrence of Salmonellosis. Although surveillance and monitoring systems are not in place and its epidemiology is not described, qualitative and quantitative syntheses of previous studies could shed light on the occurrence of the disease and the major serotypes that frequently cause infections. Therefore, this study was designed to determine the prevalence and antimicrobial resistance patterns of *Salmonella* in apparently healthy slaughtered cattle and personnel working at Jimma municipal Abattoir.

## Methods

### Study area and period

**Fig. 1:**
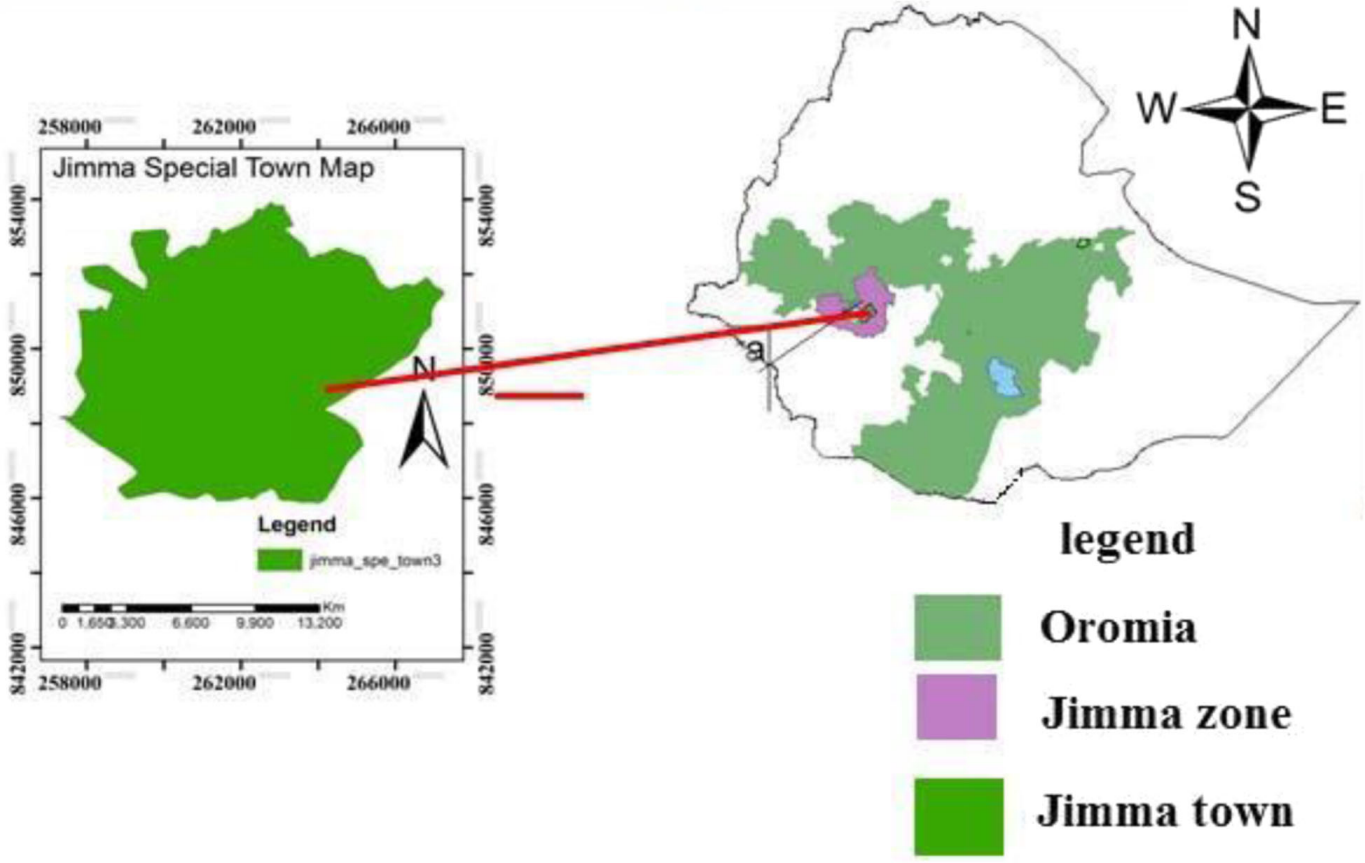
Map of the study area

The study was conducted at the Jimma municipal abattoir from May to September 2016. Jimma is located 352 km south-west of Addis Ababa at latitude of about 70 13’ – 80 56’N and longitude of about 35,052’ – 37,037’E. Jimma town has one municipality abattoir and 200 meat retailers which directly receive slaughter service from the abattoir. At the Jimma abattoir, cattle, sheep, and goats are slaughtered. Animals for slaughter are derived from different areas of the Jimma region. Daily about 30–50 cattle, 10–25 sheep, and 5–10 goat are slaughtered in this facility (Fig. 1).

At the Jimma municipal abattoir there are two Veterinary professionals and 48 trained workers assigned by the government to undertake regular slaughtering activities. Veterinarians perform anti-mortem and post-mortem inspection, and the reaming activities are carried out by trained workers.

There was no clear division of the slaughtering process into stunning, bleeding, skinning, evisceration, chilling, cutting, or frozen delivery in the Jimma municipality abattoir. Bleeding and evisceration was conducted on a horizontal position on the floor by incising the hide at the bottom of the abdomen without flying the skin. Workers hoisted the carcass manually using a chained pulley system after flying the skin and evisceration on the floor. There were no knife and axe sharpening machines. There were no means of sterilizing equipment. Carcasses were manually quartered using axes.

### Study design

A cross sectional study was conducted to find out the prevalence and antibiotic susceptibility pattern of *Salmonella* isolated from slaughter cattle and personnel working at the Jimma municipality abattoir.

### Study population

Apparently healthy cattle ready for slaughter and abattoir workers in the Jimma municipal abattoir who were engaged in slaughtering were selected randomly. Animals and abattoir personnel with clinical symptoms of salmonellosis, and also those on antibiotic treatment for at last 2 weeks prior to the study, were the exclusion criteria.

### Sample collection, isolation and identification procedure

Study samples were selected using a simple random sampling technique, and 195 slaughter cattle were sampled. Sample collection, isolation, and identification were made based on the recommendations of the International Organization for Standardization (ISO), 6579:2002 [8].

One hundred and nightly five (195) carcass swabs were collected. Each carcass was sampled on four regions, i.e., from neck, brisket, flank, and rump region. The area sampled in each region was 100 cm^2^, resulting in a total area of 400 cm^2^, using different pre-moistened commercial beef carcass sampling poly wipe kits, and the swabs were transferred to a sterile plastic cup containing 10 ml of buffered peptone water. In addition, one (1) gram of feces from the rectum of the cattle and one (1) gram of stool sample from abattoir personnel was collected and transferred into to 9 ml of buffered peptone water separately.

Homogenized carcass and fecal sample from the cattle and personnel were incubated at 37 °C. Then, 1 ml and 0.1 ml aliquot of the enrichment broths was transferred aseptically into 10 ml of Selenite Cystine and 10 ml of Rappaport–Vassiliadis with soya broth and incubated for 24 h at 37 °C and 42 °C, respectively. Following incubation, a loop full of each culture was streaked onto Brilliant Green Agar and Xylose Lysine Deoxycholate agar plates and incubated at 37 °C for 24 to 48 h. The plates (BGA and XLD) were examined for the presence of characteristics associated with *Salmonella* colonies. A single positive colony showing red color with a black center on XLD and red color on BGA agars were subjected for biochemical tests for confirmation.

### Biochemical tests

*Salmonella* isolates were identified using triple sugar iron agar, lysine iron agar, urea broth, indole test, and citrate utilization tests. These were incubated for 24 to 48 h at 37 °C. Colonies producing an alkaline slant with acid bottom and hydrogen sulfide production on TSI, positive for lysine, negative for urea hydrolysis, negative for indole test, and positive for citrate utilization were considered as *Salmonella*. Finally, susceptibility to antimicrobial was performed for all isolates.

### Antimicrobial susceptibility test

All isolates were tested by Kirby-Bauer disk diffusion method using guidelines established by the Clinical and Laboratory Standards Institute (CLSI) [12]. In brief, by taking pure isolated colonies, bacterial suspension in test tubes was adjusted and compared to 0.5McFarland turbidity standards. The diluted bacterial suspension was then transferred to a Mueller-Hinton agar plate using a sterile cotton swab and seeded uniformly by rubbing the swab against the entire agar surface and left on the bench to absorb or dry. Subsequently the selected antibiotics were placed 15-20 mm apart from each other using sterile forceps and then incubated at 37 °C for 16 to 18 h. A total of 10 selected antibiotics disks (Oxide, UK) were included: Ciprofloxacin (CIP) 5 μg, Chloramphinicol (C) 30 μg, Kanamycin (K) 10 μg, Ampicillin (AMP)10 μg, Gentamicin (GM), Tetracycline (TE) 30 μg, Sulfamethoxazole Trimethoprim (SXT) 23.75/1.25 μg, Streptomycin (S) 10 μ, Amikacin (AK) 30 μg and Cephalothin (CF) 30 μg. Finally, the zone of inhibition was measured and interprets as susceptible, intermediate, or resistant categories assigned on the basis of the critical points recommended by the CLSI.

### Data quality assurance and analysis

All the instruments used for sample processing were checked prior to the study. Proper functioning has been checked using quality control strains of *Salmonella typhimurium* (ATCC 14028) and *E.coli* (ATCC 25922). Data consistency and completeness were made all the way during data collection, data entry and analysis. Data were edited, and checked for its completeness and entered into Epi Data 3.1 then exported to Statistical Packages for Social Sciences (SPSS) version 20 for analysis.

## Results

### Socio-demographic data of Jimma municipality abattoir personnel

In the current study at the Jimma municipality abattoir; male personnel accounted for 44 (88%), Thirteen (26%) of the respondents completed primary school and 26(52%) completed high school. Thirty one (62%) respondents were in age range of 35 to 49 years. Among the participants 23(46%) had 4-8 years work experience whereas the remaining 13(26%) worked for 8 years and above. The majority the respondents 42(84%), were assigned as carcass processors depicted in Table 1.

**Table 1:** Socio-demographic data of Jimma municipality abattoir personnel from May to September 2016

Variable	Isolation of *Salmonella*		Total (%)
	Negative (%)	Positive (%)	
Sex			
Male	36(81.8)	8(18.2)	44(88)
Female	5(83.3)	1(16.7)	6(12)
Age			
18–34	15(93.8)	1(6.25)	16(32)
35–49	25(80.6)	6(19.4)	31(62)
> 50	1(33.3)	2(66.7)	3(6)
Marital status			
Married	28(80)	7(20)	35(70)
Single	13(86.7)	2(13.3)	(30)
Educational states			
1–4	9(69)	4(30.8)	13(26)
5–8	22(84.6)	4(15.4)	26(52)
9–12	8(88.9)	1(11.1)	9(18)
> Diploma	2(100)	0(0)	2(4)
Responsibility			
carcass processor	34(81)	8(19)	42(84)
meat inspector	2(100)	0(0)	2(4)
Abattoir cleaner	5(83.3)	1(16.7)	6(12)
Service year			
below1year	3(100)	0(0)	3(6)
1–3	11(100)	0(0)	11(22)
4–8 years	18(78.3)	5(21.7)	23(46)
above 8 year	9(69.2)	4(30.8)	13(26)

### Prevalence of *Salmonella* in cattle and abattoir personnel

A total of 440 samples, 195 carcass swabs, 195 cattle feces, and 50 stool samples from slaughter house personnel were collected from the Jimma municipality abattoir. From 440 samples, 42(9.5%) were positive for *Salmonella.* Of these, 22(11.3%) were detected from carcass swabs, 11(5.6%) and 9(18%) were detected from fecal samples of animal and abattoir personnel, respectively (Table 2).

**Table 2:** Prevalence of *Salmonella* in cattle and abattoir personnel in Jimma municipal abattoir from May to September 2016

Type of sample	*Salmonella*		Sample tested
	Positive (%)	Negative (%)	
Carcass swabs	22(11.3)	173(88.7)	195
Cattle Fecal	11(5.6)	184(94.4)	195
Personnel Stool	9(18)	41(82)	50
Total	42(9.5)	398(90.5)	440

### Antimicrobial resistance profiles of cattle and human isolates

Resistance to Ampicillin (54.8%), Streptomycin (42.9%), and Tetracycline (40.5%) were the most common resistance profiles identified among both human and cattle isolates. Different numbers of resistance were observed to Ciprofloxacin in both human and animal isolates (Table 3).

**Table 3:** Drug susceptibility pattern of *Salmonella* from cattle and human isolates in Jimma municipal abattoir from May to September 2016

Type of Antimicrobials Agent	Cattle isolates			Human isolates
	Carcass swabs (%)	Fecal sample (%)	Total (%)	Stool sample (%)
Ciprofloxacin (CIP)5 μg				
Susceptible	22(100)	11(100)	33(100)	9(100)
Intermediate	0(0)	0(0)	0(0)	0(0)
Resistance	0(0)	0(0)	0(0)	0(0)
Ampicillin (AMP) 10 μg				
Susceptible	9(40.9)	5(45.5)	14(42.4)	4(44.4)
Intermediate	0(0)	0(0)	0(0)	1(11.1)
Resistance	13(59.1)	6(54.5)	19(57.6)	4(44.4)
Streptomycin(S)10 μg				
Susceptible	11(50)	6(54.5)	17(51.5)	6(66.7)
Intermediate	1(4.5)	0(0)	1(3.0)	0(0)
Resistance	10(45.5)	5(45.5)	15(45.5)	3(33.3)
Chloramphenicol(C)3 μg				
Susceptible	19(86.4)	9(81.8)	28(84.8)	8(88.9)
Intermediate	0(0)	0(0)	0(0)	0(0)
Resistance	3(13.6)	2(18.2)	5(15.2)	1(11.1)
Sulfamethoxazole-Trimethoprim (SXT)23.75/1.25 μg				
Susceptible	19(86.4)	9(81.8)	28(84.8)	8(88.9)
Intermediate	0(0)	0(0)	0(0)	0(0)
Resistance	3(13.6)	2(18.2)	5(15.2)	1(11.1)
Tetracycline (T) 30 μg				
Susceptible	12(54.5)	6(54.5)	18(54.5)	6(66.7)
Intermediate	0(0)	1(9.1)	1 (3)	0(0)
Resistance	10(45.5)	4(36.4)	14(42.4)	3(33.3)
Amikacin (AK)30 μg				
Susceptible	13(59.1)	6(54.5)	19(57.6)	6(66.7)
Intermediate	2(9.1)	1(9.1)	3(9.1)	1(11.1)
Resistance	7(31.8)	4(36.4)	11(33.3)	2(22.2)
Gentamycin (GM)10 μg				
Susceptible	19(86.4)	8(72.7)	27(81.8)	9(100)
Intermediate	0(0)	0(0)	0(0)	0(0)
Resistance	3(13.6)	3(27.3	6(18.2)	0(0)
Kanamycin (K)30 μg				
Susceptible	18(81.8)	8(72.7)	26(78.8)	7(77.8)
Intermediate	0(0)	0(0)	0(0)	0(0)
Resistance	4(18.2)	3(27.3)	7(21.2)	2(22.2)
Cephalothin (CF)30 μg				
Susceptible	18(81.8)	9(81.8)	27(81.8)	5(55.6)
Intermediate	1(4.5)	0(0)	1(3)	2(22.2)
Resistance	3(13.6)	2(18.2)	5(15.2)	2(22.2)

Seventeen, (40.5%) of both human and cattle isolates indicated resistance to two or more of the antimicrobials tested, and 10/42 (23.8%) of human and cattle isolates were classified as intermediate level resistance (Table 4).

**Table 4:** Two and above antimicrobial resistance profile of human and cattle *Salmonella* isolate In Jimma municipal abattoir from May to September 2016

Number of antimicrobials to which isolate were resistance	Antimicrobial resistant pattern	Number (%)
Two	AMP,S (2)	2(11.8)
Three	AMP,S,AK(2)	4(23.5)
	AMP,S,T (1)	
	AMP,K,C (1)	
Four	AMP,S,AK,T (1)	3(17.6)
	AMP,S,GM,K (1)	
	AMP,S,T,GM (1)	
Five	AMP,S,AK,C,CF(1)	3(17.6)
	AMP,S,T,GM,K (1)	
	AMP,S,CF,T,K (1)	
Seven	AMP,S,K,T,GM,SXT, CF (1)	1(5.9)
Eight	AMP,S,AK,T,K,C,SXT, CF(2)	4(23.5)
	AMP,S,AK,T,GM,CF,C,SXT(2)	
Total		17(100)

## Discussion

In this study, *Salmonella* were isolated from 11.3% of samples collected from slaughter cattle carcasses. This finding reveled that there was a considerable rate of contamination in the Jimma slaughter house, which potentially poses a risk of causing food-associated illness. These findings support previous studies undertaken in the current study area, reporting 13.3% as *Salmonella* positive [13]. Reports from Addis Ababa indicated isolation of *Salmonella* from abdominal 9.8% and from diaphragmatic muscles 11.9% [14]. The prevalence reported in the current study is higher than other reports such as 0.0% from Egypt [15], in Namibia 0.50% [16], 4.5% in Thailand [17], 6% in Central India [18], 8.5% in Saudi Arabia [19] 4.8% [9], 7.6% [20] in Baher Dar Ethiopia. This difference possibly arises from the source of animals, types of samples, and sampling technique.

The present finding showed *Salmonella* among individuals working in the Jimma slaughter house was 9/50 (18%). This prevalence indicates that significant proportions of the study group were carriers of *Salmonella* with increased likelihood of transfer of the infection to others through contamination of food. The current finding was much higher than studies conducted elsewhere 2.4% [19], 6.0% [14], 3.4% [21], 0.93% [22] and 0.9% [23]. The possible factors that favor the transmission and prevalence of salmonellosis may include environmental and personal sanitation, socio-economic and living standards, availability of water supply, and awareness of safe food handling and preparation among individuals.

The present study also showed that 40.3% of human and cattle isolates were resistance to two or more antimicrobial drugs. This finding is in good harmony with a study conducted in Asella town on identification and antimicrobial susceptibility profiles of *Salmonella* isolated from the selected dairy farms, abattoir animals and humans, which observed 50% of isolates, were resistant to two or more antimicrobials [24]. However the present results were lower than a study conducted in Addis Ababa where 83% of isolates were resistant to two or more antimicrobials [25]. Our study showed Ciprofloxacin (100%), Chloramphenicol (85.7%), Sulphamethoxazole trimetoprime (85.7%), Gentamicin (85.7%), Kanamicin (78.6%), and Cephalothin (76.2%) have good antimicrobial activity against both human and cattle *Salmonella* isolates. This result is comparable with previous reports from animal and human isolates in Addis Ababa [25] and Asela, Ethiopia [24]. However the present findings a contradict a study conducted at the Jimma University specialized hospital which showed all *Salmonella* isolates were 100% resistant to chloramphenicol, Gentamycin and Cephalothin [26]. Another study conducted in Saudi Arabia stetted that 88.6% were resistant to Chloramphenicol [19]. The difference in *Salmonella* antimicrobial resistance levels in different areas of the country may be related to agent risk factors, such as virulence, pathogenicity, infectiousness, and host specificity associated with the genetic composition of *Salmonella* strains.

## Conclusion

The carcasses associated with the Jimma municipal abattoirs were highly contaminated with *Salmonella* and this may pose a risk to the consumer. Some asymptomatic cattle presented for slaughter contribute to carcass contamination because of *Salmonella* in the intestines that has a high chance of being transferred onto the carcass and also an 18% carriage rate of *Salmonella* species among personnel working at the Jimma municipality abattoir. This can be a possible source of salmonellosis for the community unless carriers are treated or other preventive measures are taken. A high rate of antimicrobial resistance was observed mainly to Ampicillin, Streptomycin, and Tetracycline, and both single and multiple antimicrobial resistance patterns were observed, which is of special concern in Ethiopia due to antimicrobial resistance problems. Regulatory control of antibiotic usage in livestock and humans, and periodical health checking of workers in the abattoirs, are recommended ways to minimize contamination during carcass harvesting by following sanitation procedures and implementing stricter operation laws.

## Abbreviations

BGA: Brilliant Green Agar
BPW: Buffered Peptone Water
CLSI: Clinical and Laboratory Standards Institute
MDR: Multidrug resistant
TSI: Triple Sugar Iron agar
XLD: Xylose Lysine Desoxycholate
